# Effectiveness, Cost-Utility, and Safety of Neurofeedback Self-Regulating Training in Patients with Post-Traumatic Stress Disorder: A Randomized Controlled Trial

**DOI:** 10.3390/healthcare9101351

**Published:** 2021-10-11

**Authors:** Jungtae Leem, Moon Joo Cheong, Hyeryun Lee, Eun Cho, So Young Lee, Geun-Woo Kim, Hyung Won Kang

**Affiliations:** 1 Research Center of Traditional Korean Medicine, Wonkwang University, 460, Iksan-daero, Sin-dong, Iksan 54538, Jeollabuk-do, Korea; julcho@naver.com; 2 Jangheung Integrative Medical Hospital, Wonkwang University, 121, Rohaseu-ro, Anyang-myeon, Jangheung-gun 59338, Jeollanam-do, Korea; sasayayoou@naver.com; 3 Department of Korean Neuropsychiatry Medicine, Wonkwang University, 460, Iksan-daero, Sin-dong, Iksan 54538, Jeollabuk-do, Korea; ryonghanisa@gmail.com; 4 College of Pharmacy, Sookmyung Women’s University, 100, Cheongpa-ro, Cheongpadong, Yongsan-gu, Seoul 04310, Korea; eun-cho@sookmyung.ac.kr (E.C.); soyeong511@gmail.com (S.Y.L.); 5 Department of Neuropsychiatry, Dongguk University Bundang Oriental Hospital, 268 Buljeong-ro Bundang-gu, Seongnam-si 13601, Gyeonggi-do, Korea

**Keywords:** cost-utility analysis, neurofeedback, post-traumatic stress disorder, quantitative electroencephalography, randomized controlled trial

## Abstract

Post-traumatic stress disorder (PTSD) is characterized by neurophysiological and psycho-emotional problems after exposure to trauma. Several pharmacological and psychotherapy limitations, such as adverse events and low adherence, increase the need for alternative therapeutic options. Neurofeedback is widely used for PTSD management. However, evidence of its clinical efficacy is lacking. We conducted a randomized, waitlist-controlled, assessor-blinded clinical trial to assess the effectiveness, cost-utility, and safety of 16 sessions of neurofeedback on people with PTSD for eight weeks. Eleven participants were allocated to each group. One and two subjects dropped out from the neurofeedback and control groups, respectively. The primary outcome was PTSD symptom change evaluated using the PTSD Checklist-5 (PCL-5-K). The PCL-5-K levels improved more in the neurofeedback group (44.3 ± 10.8 to 19.4 ± 7.75) than in the control group (35.1 ± 18.5 to 31.0 ± 14.92). The change value was significantly improved in the neurofeedback group (24.90 ± 13.13 vs. 4.11 ± 9.03). Secondary outcomes such as anxiety, depression, insomnia, and quality of life were also improved. In an economic analysis using EuroQol-5D, the incremental cost-per-quality-adjusted life-year was approximately $15,600, indicating acceptable cost-utility. There were no adverse events in either group. In conclusion, neurofeedback might be a useful, cost-effective, and safe intervention for PTSD management.

## 1. Introduction

Post-traumatic stress disorder (PTSD) is characterized by neurophysiological and psycho-emotional problems [1]. People with PTSD can present with specific symptoms such as hyperarousal, avoidance, and reexperiencing after exposure to trauma [2], such as a natural disaster, sexual assault, or military combat [3]. It is known that the rate of actual transition to PTSD after exposure to trauma is around 10% of all exposed patients [2]. PTSD leads to a decreased quality of life and an increased health-related resource consumption [4]. It is also known that traumatic event exposures are related to the occurrence of a number of chronic diseases [5]. The duration of PTSD symptoms affects individual life conditions [4]. Even in a case-control study, the odds ratio of suicide occurrence was about ten times higher in the PTSD group than in the normal population [6]. Moreover, a decreased health status and co-occurring mental health problems in patients with PTSD lead to increased medical costs [7]. PTSD symptoms impair personal, educational, occupational, social, and other important aspects of life [1]. Therefore, effective and appropriate management of PTSD symptoms is important from a physical, mental, and social perspective. 

As both biological and psychological factors affect the development and maintenance of PTSD, both pharmacological and psychological interventions are widely used in the management of PTSD [8]. For pharmacologic approaches, selective serotonin reuptake inhibitors (SSRIs), which have a broad effect and rapid response to PTSD, are widely used [8]. However, it is well known that long-term SSRI therapy is associated with several adverse events such as sexual dysfunction, sleep disturbance, and weight gain [9]. Psychological approaches, such as trauma-focused cognitive behavioral therapy (CBT), prolonged exposure, and cognitive processing therapy are recommended for PTSD management [3]. Unfortunately, the dropout rate of psychotherapy for PTSD is relatively high (about 16%) [10], and adverse events are not well explored [3]. Each existing pharmacologic and psychological intervention has its own strengths, indications, and limitations. No intervention guarantees complete recovery. Therefore, we still need to explore alternative options for the management of PTSD [11].

Neurofeedback is a type of biofeedback that normalizes dysregulated brain activity using audio or visual signals via electroencephalography (EEG) monitoring [2]. Various EEG components are recorded and fed to participants during the procedure so that the subject is aware of changes in brainwaves and the effect of training [12]. The aim is to regulate the amplitude of brain waves, regulate phase lag, and modify the synchrony between specific brain areas [2]. With endogenous neuromodulation (not exogenous modulation, such as transcranial magnetic stimulation), neuronal activity and connectivity can be improved, resulting in indirect behavioral change [11]. Functional and anatomical improvements, such as microstructural change and enhanced neuroplasticity, have been suggested as a possible mechanism of neurofeedback treatment [2]. Therefore, neurofeedback is a widely used non-pharmacologic intervention in various mental health conditions, including attention-deficit/hyperactivity disorder, schizophrenia, insomnia, drug addiction, and autistic spectrum disorder [12]. As neurofeedback is a non-invasive, self-regulating training that re-establishes electrophysiological activity in the brain, neurofeedback might also be useful for the management of PTSD symptoms [11]. Several studies on the use of neurofeedback for patients with PTSD are already published [11,13]. However, so far, most studies were non-randomized trials or focused on the brainwaves themselves, and not on the symptoms of PTSD [13]. Of the four randomized trials that are reported, three included only male subjects [11]. Furthermore, none of the studies adopted the PTSD Checklist (PCL-5) to assess the clinical outcomes, which evaluates the symptoms corresponding to the Diagnostic and Statistical Manual of Mental Disorders 5th Edition (DSM-5) PTSD symptom criteria.

Therefore, we designed a randomized controlled clinical trial of neurofeedback using the PCL-5 as the primary outcome, named the effectiveness, cost-utility, and safety of neurofeedback self-regulating training in patients with post-traumatic stress disorder (ESKAPE). The objective of the ESKAPE study is to explore the effectiveness, cost-utility, and safety of neurofeedback self-regulating training (NSRT) for the management of PTSD symptoms. To incorporate an intervention with proven clinical effectiveness into the healthcare system, it is necessary to evaluate whether the insurance coverage of the intervention is a sustainable change [14]. To determine this, an economic analysis is becoming an essential process for healthcare decision-making. Therefore, in this study, a cost-utility analysis was also conducted simultaneously.

## 2. Materials and Methods

We published a clinical trial protocol of the ESKAPE study [15]. The study protocol is registered at the clinical research information service (KCT0003271, https://cris.nih.go.kr/cris/search/search_result_st01_en.jsp?seq=17025&ltype=&rtype= accessed on 1 October 2021).

A detailed description of the trial process is presented in the protocol article.

### 2.1. Trial Design, Institutional Review Board Approval, and Registration

We conducted a randomized, waitlist-controlled, single-center, assessor-blinded, parallel-group clinical trial at Wonkwang University Sanbon Hospital, which is located in the city of a metropolitan area, between May 2019 and September 2020. The Institutional Review Board of Wonkwang University Sanbon Hospital approved the protocol (approval number: WMCSB 201802–10). Written informed consent was obtained from all participants before randomization.

### 2.2. Participants

#### 2.2.1. Eligibility Criteria: Inclusion Criteria

Inclusion criteria were as follows: (1) participants aged 20–55 years; (2) PTSD diagnosis according to the Structured Clinical Interview outlined in the DSM-5 (SCID-5) criteria [16]; we adopted the Korean version of the SCID-5, which is a validated, semi-structured, standard interview guide for PTSD diagnosis [17]; (3) due to a traumatic event(s) that occurred more than six months before recruitment; and (4) voluntary participation after explanation of the clinical trial.

#### 2.2.2. Eligibility Criteria: Exclusion Criteria

Exclusion criteria were as follows: (1) severe mental disease (past or recent hallucinations or delusions; manic episodes more than once; a history of alcohol dependency or abuse; or risk of suicide); (2) when it is necessary to administer continuously a substance that is judged to have an effect on the induction of symptoms, excluding concomitant drugs; (3) those with serious congenital diseases, central nervous system conditions, peripheral nervous system diseases, endocrine system diseases, immunological diseases, and/or heart, liver, and kidney conditions; (4) pregnant or lactating persons; (5) those who had participated in another clinical trial within the past month or were participating in another clinical study; (6) those that the principal investigator judged to be unable to participate.

### 2.3. Randomization, Allocation Concealment, and Blinding

A randomization table was developed by an independent statistician using block randomization methods in Microsoft Excel. The allocation ratio for the treatment and control groups was 1:1. When the consented participants met the eligibility criteria, the independent clinical research coordinator allocated the participants according to the randomization table to maintain allocation concealment. The allocated group of participants was notified of another neurofeedback practitioner. Due to the waitlist control group design, the participants and neurofeedback practitioners were not blinded. However, the assessor and statistician were blinded.

### 2.4. Intervention

#### 2.4.1. Study Schedule

**Figure 1:**
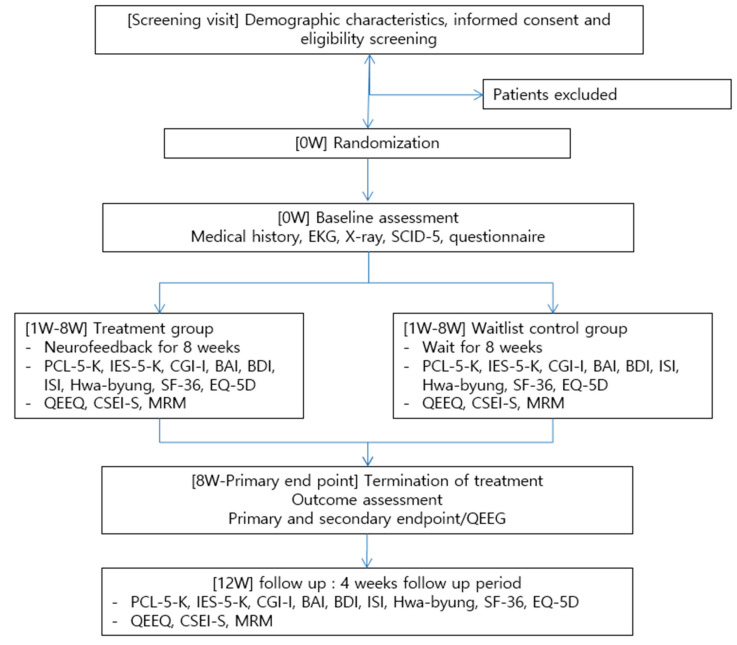
Study flowchart.

The detailed study schedule is presented in Table 1 and Figure 1.

Following visit 0 (V0), which was used for screening, the participants in the treatment group underwent 16 sessions (twice a week) of self-regulated neurofeedback training with certified neurofeedback practitioners from week one (V1) to eight (V16). The waitlist control group participants visited the hospital at V0, V1, V8, and V16 for assessment without neurofeedback training. The participants in both groups were followed up after four weeks (week 12, V17).

#### 2.4.2. Neurofeedback Procedure

A certified (Biofeedback Certification International Alliance) neurofeedback practitioner (M.J.) conducted 16 NSRT sessions based on the baseline quantitative encephalogram (QEEG) analysis. A ProComp2, 2-Channel EEG System with version 6.0 Infiniti Software (Thought Technology Ltd., Montreal West, Quebec, Canada) was used. Neurofeedback is a type of operant conditioning used to learn reinforcement and compensation via audio-feedback in an eye-closed state [18]. To reduce anxiety and stress in patients with PTSD, we utilized alpha-theta brainwave neurofeedback using the protocol suggested by Peniston and Kulkosky [19], or Smith [20]. The purpose of NSRT is to maintain a relaxed state by strengthening the alpha and theta waves and suppressing any beta waves. The condition of the participants was monitored using 24 channel electrodes. The parietal lobe (PZ) was used as the training site, and 8–12 Hz and 4–7 Hz were defined as alpha and theta waves, respectively. The 50 min training session consisted of three sets of training sessions that lasted 10 min, with a 5 min break between each training session and a 10 min finish.

#### 2.4.3. Control Group Intervention

The waitlist control group waited for 12 weeks, while the treatment group received NSRT. We allowed them to continue their usual treatment and lifestyle. For the outcome assessment, the control group participants visited the hospital at week 4 (V8 in the treatment group), 8 (V16 in the treatment group), and 12 (V17 in the treatment group).

#### 2.4.4. Concomitant Treatment

In principle, psychotherapy other than NSRT was not allowed during the trial. However, both the test and control groups were allowed to continue on any drugs or non-pharmacological treatments that were previously being taken. The acceptance criteria were as follows: (1) if the existing counseling treatment for PTSD was stably performed for at least three months before screening; (2) in the case of patients taking hypnotics, antidepressants, antipsychotics, and/or anxiolytics, the drug dose was stable for three months; (3) all drugs used to treat other diseases (high blood pressure, diabetes, hyperlipidemia, and/or pain relief) except for neurologic/neuropsychiatric diseases were permitted.

### 2.5. Primary Outcome Measures: Korean Version of the PTSD Checklist-5 (PCL-5-K) Score

The PCL-5-K uses a five-point Likert scale from zero (“not at all”) to four (“very much”) with 20 questions to measure the intensity of symptoms that participants suffered from over the previous month due to past trauma. The PTSD score ranged from 0 to 80, with higher scores indicating more severe symptoms. The cut-off value was 33 [21]. We used the validated Korean version of PCL-5-K [22]. The PCL-5-K was measured at baseline and at V8 (week 4), V16 (week 8), and V17 (week 12). The primary outcome was the difference in the change values between the treatment and control groups at V16.

### 2.6. Secondary Outcome Measures

#### 2.6.1. Impact of the Event Scale-Revised Korean Version (IES-R-K)

IES-R-K uses a five-point Likert scale from zero (“not at all”) to four (“very much”) with 22 questions that measure psychological responses such as hyperarousal, avoidance, and intrusion to life-threatening events [23]. We used the validated Korean version of the IES-R-K [24]. The cut-off value of partial PTSD was 17/18 points, and complete PTSD was 24/25 points in the Korean version. The IES-R-K was measured at baseline and at V8 (week 4), V16 (week 8), and V17 (week 12).

#### 2.6.2. Clinical Global Impression-Improvement Scale (CGI-I)

The CGI-I assesses the global improvement of participants’ symptoms after treatment compared to baseline using a seven-point Likert scale from one (very much improved) to seven (very much worse) [25]. The CGI-I was measured at V8 (week 4), V16 (week 8), and V17 (week 12).

#### 2.6.3. Beck Anxiety Inventory (BAI)

The BAI comprises 21 questions that assess the degree of anxiety using a four-point Likert scale ranging from zero to three, with a higher score indicating a severe anxiety state [26]. The Korean version of the validated BAI was used in this study. The BAI was measured at baseline and at V8 (week 4), V16 (week 8), and V17 (week 12).

#### 2.6.4. Beck Depression Inventory (BDI)

The BDI comprises 21 items that assess the degree of depression using a four-point Likert scale ranging from zero to three, with a higher score indicating a severe depressive state. A Korean version of the validated BDI was used [27]. The BDI was measured at baseline and at V8 (week 4), V16 (week 8), and V17 (week 12).

#### 2.6.5. Insomnia Severity Index (ISI)

The ISI consists of seven items that assess the severity of insomnia using a five-point Likert scale ranging from zero (no problem) to four (severe problem). An ISI score between 15 and 21 and 22–28 indicates moderate insomnia and severe insomnia, respectively [28]. The ISI was measured at baseline and at V8 (week 4), V16 (week 8), and V17 (week 12).

#### 2.6.6. Hwa-Byung Scale (HBS)

Hwa-byung is a cultural-related syndrome that means “fire disease” or “anger disease” in Korea [29]. HBS was self-reported using a Hwa-byung questionnaire that consists of 15 items about the person’s symptoms (range 0–60) and 16 items about their personality (0–64) using a five-point Likert scale from zero to four [30]. HBS was measured at baseline and at V8 (week 4), V16 (week 8), and V17 (week 12).

#### 2.6.7. Core Seven Emotions Inventory Short Form (CSEI-S)

The CSEI was developed based on the traditional Korean medicine core seven emotion theory. The seven emotions (Chiljeong in Korean) are joy, anger, thought, depression, sorrow, fear, and fright. Lee et al. (2020) developed a core seven-emotion evaluation questionnaire [31]. In our study, we used a validated Korean version of the short-form CSEI (CSEI-S), which has 28 items rated on a five-point Likert scale ranging from one (not at all) to five (very like that) [32]. The CSEI-S was measured at baseline and at V8 (week 4), V16 (week 8), and V17 (week 12). Each subscore of the CSEI-S was then analyzed.

#### 2.6.8. Mentalizing the Rooms of Mind (MRM)

MRM is a psychotherapy technique that helps patients observe their mind more objectively by structuring and visualizing the chambers of their mind, the source of their strength, and pain [33]. MRM was conducted before and after NSRT at each visit.

#### 2.6.9. QEEG Analysis

Twenty-one surface electrodes were attached to the scalp according to the International 10/20 system. Using the WinEEG (Mitsar, Russia) program, an analysis of QEEG in each band range was conducted on localization-low resolution brain electromagnetic tomography (LORETA), relative power, absolute power, and coherence. A fast Fourier transform was utilized to calculate the relative wavelength power. The filter settings were as follows: notch filter (55–65 Hz), low-frequency filter (0.3 Hz), and high-frequency filter (50 Hz).

#### 2.6.10. Safety

Adverse events (AEs) were recorded in the CRF, and their severity was categorized as mild, moderate, or severe. The causality of AEs was categorized according to the WHO-UMC criteria [34].

### 2.7. Quality of Life (QOL)

#### 2.7.1. Short Form Health Survey-36 (SF-36)

The SF-36 is the most widely used questionnaire to assess the health-related quality of life (HRQOL) and consists of 36 items with a physical component summary (PCS) and mental component summary (MCS). The PCS includes subdomains on physical functioning, bodily pain, role limitations due to physical health problems (RP), and role limitations due to personal or emotional problems (RE). The MCS includes subdomains of general mental health, social functioning, energy/fatigue or vitality (VIT), and general health perceptions (GH) [35].

#### 2.7.2. EuroQoL-5 Dimension (EQ-5D-5L)

The EQ-5D-5L evaluates HRQOL as a utility. We used the validated Korean version of the EQ-5D-5L questionnaire [36]. The EQ-5D has five dimensions: mobility, self-care, usual activities, pain/discomfort, and anxiety/depression. Each dimension is evaluated with five levels, from no problems to extreme problems. We also used the EQ-Visual Analog Scale (EQ-VAS), which assesses self-rated health status using a vertical visual analog scale.

### 2.8. Cost Outcomes

A structured questionnaire about medical and non-medical costs was administered to the CRF. Medical costs included insured medical fees and subjects’ out-of-pocket costs for health functional foods and medical devices. Subjects’ travel expenses for visiting the hospital/clinic were considered non-medical costs. Indirect costs, such as those caused by productivity loss, were investigated in terms of loss of housework hours and working hours due to anxiety symptoms or treatment. QOL and costs were measured at V1, V8, V16, and V17.

### 2.9. Sample Size Calculation

The primary outcome was the change in PCL-5-K from baseline to V16 (week eight) between the treatment and control groups. The null hypothesis was that there would be no differences between the two groups. We calculated the sample size based on the effect size (1.1) of a previous systematic review on the use of yoga in patients with PTSD [37], as the effect size acquired from the neurofeedback study was unacceptable (2.33). To calculate the sample size, we used Power Analysis and Sample Size software 2019 (NCSS, LLC. Kaysville, UT, USA, ncss.com/software/pass accessed on 1 October 2021). Each group’s calculated sample size was 19 with a 1:1 allocation ratio, 2-tailed test, two-sample *t*-test, and a test power of 90% (1- β) with a significance level of 5% (α). Considering a dropout rate of 15%, 23 participants were needed in each group.

### 2.10. Statistical Analysis

The primary outcome was the difference in the PCL-5-K change value from baseline to V16 (week eight) between the treatment and control groups. According to the normality test, the independent *t*-test or Wilcoxon rank-sum test was used. We adopted the full analysis set (FAS) method. Therefore, we included data from patients who underwent at least one follow-up measurement in the analysis. In the secondary outcome analysis, according to the normality test, we also used an independent *t*-test or Wilcoxon rank-sum test. Categorical data are presented as frequencies and ratios. Continuous data are presented as means and standard deviations. A two-tailed statistical significance value of 0.05 was adopted. AEs were presented in terms of frequency, severity, and causality. R version 4.0.3 was used for statistical analysis (R Core Team, 2020; R: a language and environment for statistical computing. R Foundation for Statistical Computing, Vienna, Austria. https://www.R-project.org accessed on 1 October 2021).

### 2.11. Economic Analysis (Cost-Utility Analysis)

We adopted a societal perspective in the economic analysis with a time horizon of 52 weeks (one year). The cost-per-quality-adjusted life-year (QALY) was the primary economic endpoint. Based on the EQ-5D scores estimated, the QOL outcome was estimated using the area under the curve [38]. We assumed that the EQ-5D value measured at V17 (week 12) could be continued until the 52nd week. All costs were measured in the Korean monetary unit won (KRW). We calculated the incremental cost-utility ratio (ICUR), which is the incremental cost to increase one QALY for the neurofeedback treatment group compared to the waitlist control group.

$$ICUR=\frac{\Delta \;Cost\;\left(neurofeedback\;group-waitlist\;control\;group\right)}{\Delta \;Increasd\;QALY\;\left(neurofeedback\;group-waitlist\;control\;group\right)}$$

## 3. Results

### 3.1. Study Flow and Patient Characteristics

**Figure 2:**
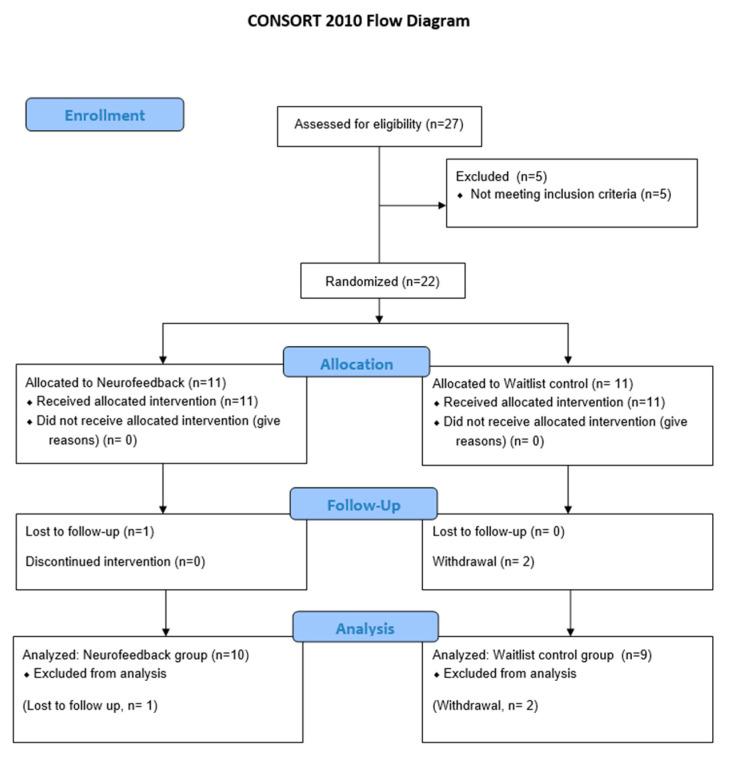
CONSORT 2010 flow diagram.

Figure 2 presents the Consolidated Standards of Reporting Trials (CONSORT) diagram regarding recruitment and the follow-up status [39].

A total of 27 participants were screened, of whom five were excluded. Twenty-two participants voluntarily agreed to provide informed consent and were allocated to the NSRT (n = 11) and waitlist control (n = 11) groups in a 1:1 ratio. In the NSRT group, PTSD was caused by domestic violence in eight participants and traffic accidents in two participants. In the wait-control group, PTSD was caused by domestic violence in eight participants and school violence in one participant. All study participants were patients whose symptoms persisted for at least 6 months according to the diagnostic criteria for PTSD. One participant in the NSRT group (dropout rate: 9.09%) and two participants in the control group (dropout rate: 18.18%) were lost to follow-up and excluded from the FAS analysis. In the initial protocol, it was estimated that a sample size of 23 people was needed in each group. However, sample recruitment had to be closed before reaching the target number of subjects due to the COVID-19 outbreak. Table 2 shows the demographic characteristics of the participants in each group. There were no statistically significant differences in the baseline characteristics between the two groups, including the primary outcome distribution (PCL-5-K).

### 3.2. Primary Outcome

As shown in Table 3, the PCL-5-K score improved from 44.3 ± 10.8 (V1) to 19.4 ± 7.8 (V16) in the NSRT group and from 35.1 ± 18.5 (V1) to 31.0 ± 14.9% in the control group. The change value at week 16 (primary outcome) between the two groups was significantly different (NSRT group: 24.90 ± 13.13 vs. waitlist control group: 4.11 ± 9.03; *p* < 0.01).

**Figure 3:**
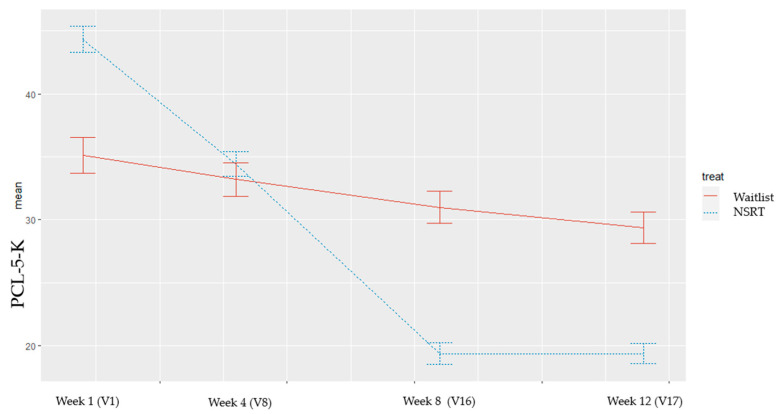
Comparison of the PCL-5-K between the treatment and control groups. The figure presents the mean with standard error represented by vertical bars. The solid red line represents the waitlist control group, and the blue dotted line represents the NSRT group.

The change trends from baseline to week 12 on the PCL-5-K score in both groups are presented in Figure 3. The PCK-5-K score change was also significant at V17 (week 12). During the four-week follow-up period, the PCK-5-K score in the treatment group did not worsen.

### 3.3. Secondary Outcome

Table 3 presents the statistically significant improvements in several outcome measures. In terms of psychological response, the change in the IES-R-K score did not differ between the two groups. In terms of the patient’s global impression of change, the change in the CGI-I score was significantly improved at weeks 4, 8, and 12. In terms of anxiety, the change in BAI significantly improved at weeks 8 and 12. In terms of depression, the change in BDI significantly improved at weeks 4, 8, and 12. In terms of insomnia, the change in the ISI score significantly improved at week eight. In terms of the Hwa-byung score, the change in the HBS score was significantly improved at weeks 8 and 12. In terms of the seven core emotions, the sub-scores of the CSEI-S (Supplementary Table S1) showed that the “joy”, “anger”, and “depression” emotion scores were not different. However, the “thought”, “sorrow”, “fear”, and “fright” emotion scores were significantly improved in the NSRT group. In the QEEG analysis, there were no statistically significant differences between the two groups at both baseline and visit 16 (Supplementary Table S2). In the qualitative MRM analysis, patients in the treatment group became more emotionally rich and focused on themselves, and they expressed general gratitude and gratitude for life.

### 3.4. AEs

There were no AEs in either group.

### 3.5. QOL

At baseline, the QOL score was lower in the NSRT group than in the waitlist control group, and the difference was statistically significant when measured with the EQ-VAS (42.80 vs. 64.67%, *p* = 0.01) and SF-36 (63.58 vs. 78.11%, *p* = 0.00). The QOL values measured in the EQ-5D, EQ-VAS, and SF-36 all increased at weeks 8 and 12 for both the NSRT and control groups (Table 3). However, the change in QOL was greater in the NSRT group than in the control group. After all the neurofeedback therapy sessions were completed, subjects’ EQ-VAS (24.00 vs. 1.44, *p* = 0.01) and SF-36 scores (21.54 vs. 5.56%, *p* = 0.01) were significantly increased from baseline compared to the control group.

### 3.6. Cost-Utility Analysis

In Table 4, the results of the cost-utility analysis are presented.

The unit of cost is the Korean Won (KRW, ₩). The AUC of the graph was plotted as the time on the x-axis and eq-5d on the y-axis. The area of the graph was then calculated.

While the direct medical costs were greater, the indirect cost in terms of productivity loss was lower for the NSRT group. The sum of the direct and indirect costs was KRW 1,684,260 (USD 1504 at an exchange rate of 1120 KRW per dollar on 28 January 2021) for the NSRT group and KRW 340,055 (USD 304) in the control group. The increments in QALY for both the NSRT and control groups were 0.122 and 0.045, respectively. Accordingly, the ICUR for the 52-week period was estimated to be approximately KRW 17,457,000 (USD 15,600) per QALY.

## 4. Discussion

### 4.1. Summary of Findings

In this study, we found that 16 sessions of NSRT over eight weeks effectively reduced the PCL-5-K symptom score in patients with PTSD. The effect persisted for one month of follow-up. NSRT also reduced anxiety, depression, and insomnia in patients with PTSD. Subjective patient perception of disease status and QOL were also better in the NSRT group. The Hwa-byung score also improved in the NSRT group. No AEs were reported in either group. QOL values significantly increased in the NSRT group. In the economic analysis, the cost-utility outcome of NSRT therapy was acceptable. Therefore, we found NSRT to be effective, cost-effective, and safe for the management of patients with PTSD. NSRT appears to be a complementary treatment that can be used in addition to conventional medicine for the management of patients with PTSD. However, since this study has several limitations, and the sample size is too small, additional research is needed to confirm the results.

### 4.2. Debates

According to recent systematic reviews of neurofeedback on PTSD, several clinical studies have been conducted [2,11,13]. However, most clinical studies are non-randomized clinical trials. In addition, the participants were biased toward males, especially in studies on war veterans. Previous research focused more on physiological responses and EEG than on the improvement of clinical symptoms. However, in our study, we focused more on the clinical effectiveness on symptom change, safety, adherence, and cost-utility to help stakeholders make decisions.

Several underlying mechanisms of NSRT for PTSD symptoms were revealed in previous studies, such as modulating neuroplasticity, improving the connectivity of neurocognitive networks, and resulting in microstructural changes [2,11,13]. NSRT regulates electrophysiological activity in the brain via endogenous neuromodulation, which induces neuronal activity/connectivity changes that can control a person’s behavior [40]. NSRT may produce connectivity changes at both the synaptic and circuitry level [2]. In previous imaging studies, sustained attention caused by repeated NSRT sessions led to microstructural changes in the gray and white matter [41]. With respect to the default mode network, NSRT training significantly reduced the alpha amplitude, which is related to the resting state [40]. Based on these functional and structural effects, it is presumed that NSRT affects the neurocognitive network involved in PTSD pathology [2].

In terms of the minimal clinically important difference (MCID), the effect size (Cohen’s d) of NSRT on PCL-5-K in our study was about 1.8 (large effect). In a previous report on PCL, which consisted of 17 self-reported items corresponding to the DSM-4, a 20-point change was suggested as clinically significant [42]. In another MCID study on PCL [43], the Z-score change ranged between 0.5 and 0.8 standard deviations and was regarded as clinically meaningful. Although the MCID of PCL-5 has yet to be studied, we anticipate that there will not be a significant difference in the MCID. In a non-pharmacologic intervention (repetitive transcranial magnetic stimulation) study for major depression, the estimated placebo effect of non-pharmacologic interventions was 0.82 (95% CI, 0.63 to 1). As the effect size of our research is large enough considering the placebo effect, we suggest that the results of our study can be assumed to have sufficient clinical significance as well as statistical significance.

In our study, several secondary clinical outcomes were improved. Anxiety and depression levels improved and remained unchanged during the follow-up period. The severity of insomnia also improved. In patients with PTSD, 70–91% suffer from insomnia. Furthermore, insomnia has a negative effect on symptom severity, QOL, and daily functioning [44]. Our data suggest that insomnia severity improved without additional interventions, except for NSRT. Therefore, the improvement of QOL in the NSRT group might be a natural result considering the improvement in sleep status. The patient’s subjective perception of improvement, as evaluated by the CGI-I, also improved. The Hwa-byung score, which reflects the prevalence of a culture-bound anger syndrome, also improved in our study [29]. The relationship between PTSD and Hwa-byung has yet to be explored. Our data will be utilized in further research on PTSD and Hwa-byung. Our study found that NSRT is effective not only for PTSD symptoms but also for anxiety, depression, insomnia, subjective perception, and QOL. The CSEI-S total score did not significantly change; however, some emotions possibly related to trauma, such as thought, sorrow, fear, and fright, were improved. In contrast, joy, anger, and depression did not significantly change. Although the BDI score improved, the depression sub-score in the CSEI-S did not change. This suggests that depression in the CSEI-S questionnaire [32], which measures depression in terms of traditional medicine, might not mean the same thing as depression in modern disease. Further research is required on this topic.

Since psychological therapy requires several continuous sessions for effective PTSD management, a stable psychological intervention that can be applied safely and in the long term is required. In terms of adherence, 9% (1/11) of the participants were excluded from the NSRT group. Even though the sample size was relatively small, it was smaller than the dropout rate (16%) in a previous study on psychotherapy in a PTSD trial [10]. Unlike trauma-focused therapy such as CBT, eye movement desensitization, and reprocessing, a treatment that focuses on trauma symptom reduction approaches such as NSRT might have a lower dropout rate than trauma-focused therapy [10]. AEs were not reported in the present study. AEs from psychotherapy in PTSD are not well established, which increases the risk of long-term treatment [3]. In a previous psychotherapy study, the number of sessions attended was found to be a predictor of PTSD symptom improvement [45], indicating that higher adherence leads to better outcomes. In terms of adherence and AEs, NSRT might be a valuable option for the long-term treatment of PTSD symptoms.

Our study results somewhat differed from those of previous RCTs [19,46,47,48]. Three studies included only men [19,46,48], and two of these studies included only veterans [19,46]. In this study, not only war-induced forms of PTSD, but various other types of PTSD that may occur in daily life, were investigated to determine the effect of NSRT. Therefore, unlike existing RCTs that only targeted men, women were also included. In one RCT that included women [47], the proportion of women was 75%, but it was difficult to make a direct comparison because the measured outcome variables were different from those in our study. However, as a result of a meta-analysis of the improvement of PTSD symptoms measured through various clinical outcomes in previous RCTs [11], the derived standardized mean difference (SMD) was approximately 2.3, which indicates a large effect. Since the effect size derived from our study was approximately 1.8, it was considered to show some consistent results. The effect of NSRT according to the participants’ sex or disease severity may differ in clinical practice. Since a significant number of participants in this study were patients with PTSD due to domestic violence, there is a possibility that different results may be obtained when NSRT is applied to clinical practice for men who are veterans. This requires additional research through clinical trials and meta-analysis methodologies in the future. In a previous study [19], medication usage was measured; however, the usage was not measured separately, as the medication was administered without alterations during the clinical trial period. Since the dose change of a drug is generally made slowly, it is considered appropriate to design a longer clinical trial period to observe the effect related to the dosage of the drug.

In a previous report, the medical costs of patients with PTSD were twice as high as those of the normal population [7]. Therefore, cost-utility is an important issue in the management of PTSD. In the present study, the incremental cost of NSRT therapy was KRW 17,457,000 (USD 15,600) per QALY compared to the waitlist control group. Considering that the ICUR value of up to twice the per capita gross domestic profit (GDP; the GDP per capita in Korea was USD 31,838.2 in 2019) is usually regarded as cost-effective by reimbursement decision makers in the Korean National Health Insurance Scheme [49], the ICUR of NSRT, which is about half the value of the GDP per capita (USD 31,832), might be judged as cost-effective.

The disease burden of PTSD increases with a lower QOL in patients [3]. In addition, medical costs increase by about 142% in the year after the diagnosis of PTSD [7]. Considering the significantly increased QOL and lower ICUR of NSRT shown in the present study, it is suggested that the application of NSRT therapy for patients with PTSD in the early phase might be a reasonable and cost-effective alternative.

### 4.3. Strengths, Limitations, and Implications for Further Research

Our study had several strengths. Most NSRT trials for patients with PTSD focus only on an EEG analysis and not on the clinical outcomes of PTSD. However, our study examined a number of clinical outcomes, such as anxiety, depression, insomnia, QOL, and PTSD symptoms. We also conducted a cost-utility analysis in our trial, which could be useful for stakeholders’ decisions on National Health Insurance coverage. Our study has several limitations. The most important limitation was that the sample size was relatively small which might have exaggerated the effect size. In our study, the estimated effect size was relatively larger than that reported in other studies. The effect size may be smaller in a larger clinical trial. Therefore, caution should be exercised when adopting the effect size directly in further clinical practice and research. Next, because of COVID-19, the planned number of patients could not be recruited, and the study had to be terminated early. We could not acquire treatment response data for the waitlist control group after the completion of the waiting period due to the COVID-19 pandemic. We recruited 50% of the planned number of participants. Further studies with a larger number of participants are required. The follow-up period was relatively short. In future studies, it is recommended to design a protocol that has a longer follow-up period. As mentioned above, this is also related to the robustness of the cost-utility analysis. Next, we could not adopt a placebo-controlled design. However, it is difficult to apply a placebo-controlled design in a NSRT trial. We believe that a waitlist control design or head-to-head design is the best possible alternative for the control group in NSRT trials. In brief, studies with longer follow-up periods and more participants are required. Alternatively, an indirect head-to-head comparison of possible interventions using a network meta-analysis design could be conducted. We are also preparing a separate qualitative analysis article on MRM in this trial.

Some limitations to our economic analyses must also be acknowledged. As economic data were collected during the ongoing clinical trial, the length of follow-up was relatively short compared to the stand-alone economic study. Thus, the utility until the 52nd week was assumed to be maintained from the utility value of the week 12, which was measured four weeks after the last treatment. The study participants, sample size, and number of sites were also limited in the present study, which leads to increased uncertainty in the outcome estimates of utility, QALY, and costs. In addition, a sensitivity analysis was not conducted because of the limited available data in the trial, which might hamper the robustness of the economic analysis.

In future studies, we recommend a placebo-controlled or head-to-head comparison design with a long-term follow-up (>1 year) using telephone or health insurance data. This will provide the possibility to explore the cost-utility analysis results, which are reanalyzed based on clinical data that have been followed for more than one year. In addition, we propose to design a study that provides more generalizable data by recruiting a larger number of participants with various types of PTSD. 

## 5. Conclusions

In our study, 16 sessions of NSRT over eight weeks effectively reduced PCL-5-K PTSD symptoms compared to the waitlist control group. NSRT reduced anxiety, depression, and insomnia in patients with PTSD and improved the QOL and subjective perception of PTSD severity. Moreover, no AEs were reported in the NSRT group. Considering the direct and indirect costs, NSRT treatment was found to be cost-effective in increasing QALY for patients with PTSD in Korea. Taken together, NSRT is a useful, cost-effective, and safe intervention for patients with PTSD.

## Figures and Tables

**Table 1:** Study Schedule.

Assessment	Enrollment	Treatment Phase			Follow-Up Phase
	Screening	Before V1 (Week One)	After V8 (Week Four)	After V16 (Week Eight)	V17 (Week 12)
Informed consent	X				
Demographic characteristics	X				
Medical history	X				
Vital signs and physical examination	X	Every visit before the intervention			
EKG and X-ray	X				
Blood * and urine test	X				
SCID-5	X				
Inclusion/exclusion criteria	X				
Jing Ji and Zheng Chong		X			
Mibyeong		X			
KS-15		X			
KSRI-SF		X			
PCL-5-K (primary outcome)		X	X	X ^#^	X
IES-R-K		X	X	X	X
CGI-I		X	X	X	X
BAI		X	X	X	X
BDI		X	X	X	X
ISI		X	X	X	X
Hwa-byung		X	X	X	X
SF-36		X	X	X	X
EQ-5D, EQ-VAS		X	X	X	X
Cost		X	X	X	X
CSEI-S		Every visit before and after the intervention			
MRM		Every visit before and after the intervention			
QEEG		X		X	X
Safety assessment		During the trial, including waiting and follow-up period			

# Time point for analyzing the primary outcome. If necessary, unscheduled visits will be allowed and recorded in the medical record and care report form. * Blood test: red blood cells, white blood cells, hemoglobin, hematocrit, platelets, erythrocyte sedimentation rate, C-reactive protein, aspartate aminotransferase, alanine aminotransferase, gamma-glutamyl transferase, alkaline phosphatase, total bilirubin, glucose, blood urea nitrogen, creatinine, electrolytes (Na, K, Cl), total protein, albumin, and thyroid function test (T3, TSH, free T4). Women of childbearing age will be further tested for urine hCG to identify pregnancy before the first treatment. A three-day window was allowed for each visit.

**Table 2:** Baseline characteristics of participants.

Characteristic	Experiment (N = 10)	Wait-Control (N = 9)	*p*-Value
Sex (male)	1 (10%)	1 (11.1%)	0.93
Age (years)	44.40 ± 13.61	43.56 ± 19.10	0.26
Height (centimeter)	160.20 ± 7.41	160.56 ± 6.21	0.35
Weight (kilogram)	60.66 ± 11.68	56.67 ± 8.73	0.31
Pulse (beat per min)	79.20 ± 7.96	79.78 ± 12.51	0.66
SBP (mmHg)	121.0 ± 13.1	123.3 ± 14.4	0.63
DBP (mmHg)	77.4 ± 10.7	77.9 ± 15.3	0.90
Origin of PTSD	Domestic violence (8 participants) Traffic accident (2 participants)	Domestic violence (8 participants) School violence (1 participant)	NA
IES-R-K	47.50 ± 16.78	34.56 ± 16.90	0.16
PCL-5-K total score	44.30 ± 10.87	35.11 ± 18.54	0.52

All values are the mean ± standard deviation except for sex.

**Table 3:** Mean difference of the NSRT group versus the waitlist control group in terms of the clinical outcome variables at V1, V8, V16, and V17.

Outcome Variables	NSRT Group (N = 10)	Waitlist Control Group (N = 9)	*p*-Value
PCL-5-K (^#^ Primary outcome, V16)			
V1 (Baseline)	44.3 ± 10.8	35.1 ± 18.5	0.31
V8 (week 4)	34.43 ± 9.51	33.2 ± 16.35	
Difference (V1–V8)	−9.9 ± 9.84	−1.89 ± 7.39	0.10
V16 (week 8)	19.4 ± 7.75	31.0 ± 14.92	
Difference (V1–V16) ^#^	−24.90 ± 13.13	−4.11 ± 9.03	<0.01 *
V17 (week 12)	19.4 ± 6.52	29.4 ± 13.99	
Difference (V1–V17)	−24.90 ± 14.50	−5.67 ± 12.91	0.01 *
IES-R-K			
V1 (Baseline)	47.50 ± 16.78	34.56 ± 16.90	0.16
V8 (week 4)	40.30 ± 13.99	33.44 ± 19.74	
Difference (V1–V8)	7.20 ± 22.26	1.12 ± 8.55	0.21
V16 (week 8)	25.60 ± 10.47	31.11 ± 16.61	
Difference (V1–V16)	21.90 ± 22.48	3.45 ± 12.03	0.05
V17 (week 12)	23.50 ± 11.77	28.22 ± 18.89	
Difference (V1–V17)	24.00 ± 26.60	6.34 ± 11.73	0.10
CGI-I			
V8 (week 4)	3.10 ± 0.0.32	3.89 ± 0.33	<0.01 *
V16 (week 8)	2.70 ± 0.67	4.22 ± 0.44	<0.01*
V17 (week 12)	2.30 ± 0.48	3.78 ± 0.67	<0.01 *
BAI			
V1 (Baseline)	48.80 ± 8.74	43.22 ± 16.24	0.55
V8 (week 4)	42.20 ± 10.22	41.22 ± 9.68	
Difference (V1–V8)	6.60 ± 6.06	2.00 ± 8.28	0.24
V16 (week 8)	32.20 ± 8.50	39.44 ± 9.66	
Difference (V1–V16)	16.60 ± 7.20	3.78 ± 9.67	0.00 *
V17 (week 12)	32.20 ± 5.73	40.22 ± 10.22	
Difference (V1–V17)	16.60 ± 6.83	3.00 ± 10.11	0.00 *
BDI			
V1 (Baseline)	26.10 ± 7.87	16.89 ± 11.13	0.03 *
V8 (week 4)	17.00 ± 5.50	15.56 ± 8.80	
Difference (V1–V8)	9.10 ± 4.61	1.33 ± 7.89	0.02 *
V16 (week 8)	10.40 ± 5.72	14.89 ± 10.95	
Difference (V1–V16)	15.70 ± 7.47	2.00 ± 3.94	0.00 *
V17 (week 12)	11.00 ± 5.16	15.89 ± 10.37	
Difference (V1–V17)	15.10 ± 8.89	1.00 ± 5.20	0.00 *
ISI			
V1 (Baseline)	17.30 ± 5.96	12.11 ± 6.89	0.11
V8 (week 4)	14.60 ± 4.84	11.44 ± 5.64	
Difference (V1–V8)	2.70 ± 5.70	0.78 ± 3.42	0.28
V16 (week 8)	9.40 ± 4.58	11.78 ± 5.56	
Difference (V1–V16)	7.90 ± 7.43	0.44 ± 3.97	0.02 *
V17 (week 12)	9.60 ± 5.99	9.89 ± 6.99	
Difference (V1–V17)	7.70 ± 5.96	2.33 ± 4.27	0.05
HBS			
V1 (Baseline)	70.2 ± 11.1	55.0 ± 23.4	0.10
V8 (week 4)	62.9 ± 9.48	57.78 ± 26.63	
Difference (V1–V8)	−7.30 ± 9.17	2.78 ± 9.82	0.08
V16 (week 8)	52.1 ± 13.2	53.78 ± 21.71	
Difference (V1–V16)	−18.10 ± 14.65	−1.22 ± 6.55	0.00 *
V17 (week 12)	52.3 ± 11.0	51.22 ± 19.29	
Difference (V1–V17)	−17.90 ± 12.57	−3.78 ± 10.22	0.03 *
EQ-5D			
V1 (Baseline)	0.61 ± 0.21	0.76 ± 0.09	0.75
V8 (week 4)	0.71 ± 0.13	0.81 ± 0.18	0.19
Difference (V1–V8)	0.09 ± 0.18	0.05 ± 0.15	0.54
V16 (week 8)	0.75 ± 0.20	0.80 ± 0.15	0.56
Difference (V1–V16)	0.13 ± 0.13	0.04 ± 0.12	0.12
V17 (week 12)	0.74 ± 0.16	0.81 ± 0.13	0.35
Difference (V1–V17)	0.13 ± 0.13	0.05 ± 0.11	0.15
EQ-VAS			
V1 (Baseline)	42.80 ± 18.70	64.67 ± 8.93	0.01 *
V8 (week 4)	59.90 ± 15.37	60.33 ± 10.42	0.94
Difference (V1–V8)	17.10 ± 15.30	−4.33 ± 9.53	0.00 *
V16 (week 8)	66.80 ± 9.95	66.11 ± 12.44	0.90
Difference (V1–V16)	24.00 ± 20.11	1.44 ± 12.99	0.01 *
V17 (week 12)	70.80 ± 13.57	68.33 ± 13.92	0.70
Difference (V1–V17)	28.00 ± 16.12	3.67 ± 12.64	0.00 *
SF-36			
V1 (Baseline)	63.58 ± 5.88	78.11 ± 10.88	0.00 *
V8 (week 4)	72.80 ± 11.25	83.65 ± 10.51	0.05 *
Difference (V1–V8)	9.22 ± 9.77	5.54 ± 13.28	0.50
V16 (week 8)	85.12 ± 13.30	83.67 ± 9.41	0.79
Difference (V1–V16)	21.54 ± 13.17	5.56 ± 12.85	0.01 *
V17 (week 12)	84.60 ± 11.43	87.11 ± 11.15	0.64
Difference (V1–V17)	21.02 ± 13.97	9.00 ± 12.16	0.06

* Statistically significant (*p* < 0.05). ^#^, Time point for analyzing the primary outcome. V1, Visit 1; V8, Visit 8; V16, Visit 16; V17, Visit 17. Data are expressed as the mean ± standard deviation. As data were not normally distributed, a non-parametric analysis (Wilcoxon rank-sum test) was adopted. All p-values were acquired from non-parametric analyses. However, to help readers intuitively understand the data, the values are presented using the mean and standard deviation instead of the median and interquartile range.

**Table 4:** Cost, utility, and economic analysis.

	Neurofeedback Group (N = 10)	Waitlist Control Group (N = 9)
Cost (KRW)		
Neurofeedback training fee	1,280,000	0
Medical cost	220,253	120,667
Transportation expense	67,462	67,462
Loss of productivity	116,545	151,926
Total cost (8 weeks)	1,684,260 (A)	340,055 (B)
Area under the curve (AUC) of utility (EQ-5D)		
Week 0–4	0.190	0.094
Week 5–8	0.454	0.168
Week 9–12	0.524	0.170
Week 13–52	5.200	1.920
Increased QALY		
AUC total/one year (52 weeks)	0.122 (C)	0.045 (D)
ICUR: (A–B)/(C–D)		
17,457,208 (KRW/QALY)		

## Data Availability

Not applicable.

## Supplementary Materials

The following are available online at https://www.mdpi.com/article/10.3390/healthcare9101351/s1, Table S1: Mean difference for neurofeedback versus the waitlist control group on the subscale of CSEI-S variables at V1, V8, V16, and V17, Table S2: QEEG data.
