# Supplementary material for: Effectiveness, Cost-Utility, and Safety of Neurofeedback Self-Regulating Training in Patients with Post-Traumatic Stress Disorder: A Randomized Controlled Trial
Source: Healthcare (Basel). 2021 Oct 11;9(10):1351. doi: 10.3390/healthcare9101351 (PMC8544423; doi:10.3390/healthcare9101351)
Supplement: Supplementary file 1 [file healthcare-09-01351-s001.zip › healthcare-1397051-supplementary.pdf]

**Supplementary Legends**

**Supplementary Table 1**

**Mean difference for neurofeedback versus the waitlist control group on the subscale of CSEI-S variables at V1, V8, V16, and V17.**

**Supplementary Table 2**

**QEEG data**

**2.1 NSRT Group, Closed eye, baseline**

**2.2 Waitlist Control Group, Closed eye, baseline**

**2.3 NSRT Group, opened eye, baseline**

**2.4 Waitlist control Group, opened eye, baseline**

**2.5 NSRT Group, Closed eye, Visit 16**

**2.6 Waitlist Control Group, Closed eye, Visit 16**

**2.7 NSRT Group, opened eye, Visit 16**

**2.8 Waitlist control Group, opened eye, Visit 16**

**Supplementary Table 1.** Mean difference for neurofeedback versus the waitlist control group on the subscale of CSEI-S variables at V1, V8, V16, and V17.

| | Neurofeedback group (N=10) | Waitlist control group (N=9) | p-value |
|---|---|---|---|
| **CSEI-S (total)** | | | |
| V1 (Baseline) | 77.90±15.46 | 71.00±19.07 | .40 |
| V17 (week 12) | 56.50±12.64 | 68.67±13.12 | |
| Difference (V1-V17) | 21.40±15.02 | 2.33±12.66 | .13 |
| **CSEI-S (joy)** | | | |
| V1 (Baseline) | 9.10±2.92 | 10.67±3.04 | .28 |
| V17 (week 12) | 10.3±3.77 | 9.67±3.28 | |
| Difference (V1-V17) | -1.20±3.26 | 1.00±2.60 | .08 |
| **CSEI-S (anger)** | | | |
| V1 (Baseline) | 9.50±3.98 | 7.56±3.24 | 1.00 |
| V17 (week 12) | 6.50±1.65 | 6.89±2.26 | |
| Difference (V1-V17) | 3.00±2.98 | 0.67±1.87 | .05 |
| **CSEI-S (thought)** | | | |
| V1 (Baseline) | 13.10±3.03 | 13.56±2.46 | .06 |
| V17 (week 12) | 9.40±2.76 | 12.89±3.33 | |
| Difference (V1-V17) | 3.70±3.83 | 0.67±2.55 | .00* |
| **CSEI-S (depression)** | | | |
| V1 (Baseline) | 10.20±2.53 | 7.78±2.68 | .28 |
| V17 (week 12) | 6.70±2.45 | 8.56±2.96 | |
| Difference (V1-V17) | 3.50±3.72 | -0.78±2.39 | .07 |
| **CSEI-S (sorrow)** | | | |
| V1 (Baseline) | 12.30±3.59 | 10.33±4.36 | .11 |
| V17 (week 12) | 7.60±2.59 | 8.78±2.54 | |
| Difference (V1-V17) | 4.70±2.87 | 1.56±3.57 | .00* |
| **CSEI-S (fear)** | | | |
| V1 (Baseline) | 12.00±2.79 | 9.44±4.67 | .84 |
| V17 (week 12) | 8.00±3.13 | 9.56±3.50 | |
| Difference (V1-V17) | 4.00±3.09 | -0.11±2.03 | .00* |
| **CSEI-S (fright)** | | | |
| V1 (Baseline) | 11.70±3.95 | 11.67±5.85 | .55 |
| V17 (week 12) | 8.00±4.45 | 12.33±4.39 | |
| Difference (V1-V17) | 3.70±2.31 | -0.67±3.32 | .01* |

*, statistically significant ($p<0.05$)

## Supplementary Table 2 QEEG data

## Supplementary Table 2.1 NSRT Group, Closed eye, baseline

| Experimental group (Eye Closed) (N=10) | | | | | | |
|---|---|---|---|---|---|---|
| Location | Delta | Theta | Alpha | Beta | High Beta | Gamma |
| FP1-LE | 14.14±7.69 | 7.65±4.03 | 21.46±21.29 | 6.28±3.42 | 1.06±0.57 | 1.57±1.27 |
| FP2-LE | 14.78±8.21 | 8.09±4.39 | 21.66±21.88 | 6.79±4.45 | 1.19±0.99 | 1.52±1.16 |
| F3-LE | 14.06±6.59 | 13.48±9.52 | 31.30±31.20 | 8.77±6.04 | 1.18±0.61 | 1.53±1.25 |
| F4-LE | 14.54±6.69 | 15.23±12.00 | 31.08±29.42 | 9.28±7.06 | 1.16±0.71 | 1.46±0.97 |
| C3-LE | 13.39±7.27 | 15.37±17.57 | 31.37±26.85 | 9.77±6.72 | 1.15±0.73 | 1.12±0.65 |
| C4-LE | 13.03±6.80 | 17.49±24.83 | 29.10±22.52 | 9.90±6.26 | 1.12±0.63 | 1.22±0.75 |
| P3-LE | 12.64±6.93 | 17.48±28.05 | 33.79±29.14 | 12.43±10.27 | 1.02±0.57 | 0.92±0.41 |
| P4-LE | 13.22±7.22 | 17.89±28.98 | 36.28±30.94 | 11.93±8.24 | 1.01±0.49 | 0.92±0.42 |
| O1-LE | 11.77±7.33 | 14.11±21.82 | 40.26±30.33 | 12.25±8.82 | 0.93±0.45 | 0.99±0.48 |
| O2-LE | 11.01±7.20 | 10.89±11.56 | 38.82±33.26 | 11.66±7.60 | 0.93±0.42 | 0.98±0.45 |
| F7-LE | 15.56±7.26 | 6.95±3.34 | 16.88±16.90 | 5.72±3.28 | 0.92±0.60 | 1.22±0.84 |
| F8-LE | 15.28±7.50 | 7.61±4.52 | 15.50±14.75 | 6.16±4.53 | 0.92±0.65 | 1.28±1.03 |
| T3-LE | 9.67±7.66 | 5.87±5.13 | 9.95±7.88 | 5.96±4.86 | 0.91±0.75 | 1.62±1.66 |
| T4-LE | 8.80±7.12 | 6.88±9.23 | 8.97±6.56 | 6.11±6.92 | 1.05±1.32 | 1.63±2.06 |
| T5-LE | 9.51±6.49 | 11.53±17.90 | 24.31±28.16 | 8.33±6.31 | 0.78±0.43 | 0.84±0.45 |
| T6-LE | 11.35±8.36 | 12.07±19.71 | 28.46±20.56 | 8.17±4.97 | 0.86±0.54 | 0.97±0.74 |
| Fz-LE | 15.22±6.63 | 16.25±11.96 | 35.99±35.48 | 9.28±6.14 | 1.13±0.52 | 1.42±1.20 |
| Cz-LE | 15.72±6.52 | 20.27±23.08 | 40.17±34.34 | 10.69±6.77 | 1.18±0.58 | 1.15±0.47 |
| Pz-LE | 14.59±7.02 | 20.52±32.65 | 41.53±36.16 | 12.30±8.84 | 1.03±0.49 | 0.93±0.38 |

## Supplementary Table 2.2 Waitlist Control Group, Closed eye, baseline

| Waitlist control group (Eye Closed) (N=9) | | | | | | |
|---|---|---|---|---|---|---|
| Location | Delta | Theta | Alpha | Beta | High Beta | Gamma |
| FP1-LE | 42.68±66.4 | 13.18±15.48 | 24.04±12.99 | 7.30±2.33 | 1.41±0.84 | 2.02±1.45 |
| FP2-LE | 41.05±61.19 | 12.87±13.41 | 24.53±13.63 | 8.23±3.72 | 1.96±2.00 | 3.09±3.54 |
| F3-LE | 20.10±11.15 | 12.90±4.60 | 33.26±16.58 | 10.60±3.66 | 1.79±0.86 | 2.01±0.82 |
| F4-LE | 19.92±11.26 | 12.95±4.63 | 33.52±19.25 | 10.43±4.10 | 1.67±0.94 | 1.92±0.94 |
| C3-LE | 16.74±6.87 | 11.83±4.59 | 34.89±16.94 | 15.13±9.32 | 2.05±1.06 | 2.24±1.29 |
| C4-LE | 17.50±11.82 | 11.11±4.89 | 34.56±19.79 | 13.58±8.30 | 1.62±0.87 | 1.61±0.56 |
| P3-LE | 15.27±6.04 | 11.31±4.86 | 47.26±40.54 | 17.75±12.42 | 1.61±1.01 | 1.89±1.62 |
| P4-LE | 15.85±8.25 | 11.64±7.00 | 53.28±74.43 | 15.14±8.92 | 1.31±0.64 | 1.39±0.65 |
| O1-LE | 16.27±9.70 | 12.20±6.11 | 73.31±89.56 | 16.20±11.06 | 1.30±0.51 | 1.71±0.88 |
| O2-LE | 12.51±5.90 | 9.86±4.98 | 48.92±66.78 | 12.38±7.09 | 1.21±0.59 | 1.66±1.00 |
| F7-LE | 17.36±13.05 | 7.71±2.91 | 18.33±8.61 | 7.30±3.15 | 1.74±1.52 | 2.47±1.97 |
| F8-LE | 15.25±14.23 | 6.92±3.63 | 16.70±10.26 | 6.00±2.54 | 1.14±0.59 | 1.69±1.16 |
| T3-LE | 9.59±3.63 | 5.63±1.79 | 12.79±4.68 | 9.84±6.73 | 1.99±2.69 | 3.57±5.00 |
| T4-LE | 8.83±4.21 | 5.00±2.53 | 12.04±6.24 | 6.45±2.79 | 1.04±0.61 | 1.74±1.54 |
| T5-LE | 11.31±3.77 | 8.27±3.18 | 30.95±23.11 | 11.87±6.54 | 1.62±1.91 | 2.36±3.44 |
| T6-LE | 14.75±13.91 | 10.48±10.64 | 57.31±90.98 | 12.47±10.99 | 1.30±0.84 | 1.87±1.46 |
| Fz-LE | 23.72±12.92 | 15.83±5.35 | 39.46±20.08 | 15.01±13.82 | 4.07±7.33 | 9.54±23.93 |
| Cz-LE | 19.95±7.85 | 14.63±5.17 | 44.00±24.40 | 13.92±7.66 | 2.32±1.70 | 1.75±0.63 |
| Pz-LE | 17.15±7.62 | 12.95±5.85 | 56.60±49.68 | 16.25±10.24 | 1.45±0.70 | 1.46±0.63 |

## Supplementary Table 2.3 NSRT Group, opened eye, baseline

| Experimental group (Eye Opened) (N=10) | | | | | | |
|---|---|---|---|---|---|---|
| Location | Delta | Theta | Alpha | Beta | High Beta | Gamma |
| FP1-LE | 67.67±69.90 | 11.11±6.89 | 9.99±11.56 | 7.10±3.07 | 1.90±0.94 | 2.59±1.61 |
| FP2-LE | 80.30±98.74 | 14.02±14.10 | 10.98±12.49 | 8.67±7.23 | 2.35±2.20 | 3.20±3.27 |
| F3-LE | 22.93±17.49 | 11.62±7.34 | 13.93±16.22 | 7.74±5.32 | 1.65±1.14 | 2.50±2.21 |
| F4-LE | 22.10±15.66 | 13.20±10.08 | 15.29±18.47 | 8.69±7.50 | 1.93±1.93 | 2.89±3.18 |
| C3-LE | 13.82±5.89 | 13.02±15.17 | 15.78±21.65 | 7.45±4.52 | 1.14±0.57 | 1.35±0.86 |
| C4-LE | 13.41±5.14 | 14.83±20.45 | 16.34±21.45 | 7.98±4.17 | 1.22±0.52 | 1.64±1.15 |
| P3-LE | 12.00±3.94 | 14.33±21.30 | 20.92±31.28 | 8.75±7.03 | 0.93±0.41 | 0.97±0.34 |
| P4-LE | 12.45±5.01 | 13.56±19.05 | 22.19±33.50 | 8.57±5.54 | 0.93±0.35 | 1.04±0.39 |
| O1-LE | 10.92±4.03 | 10.32±12.56 | 25.50±48.05 | 8.39±5.47 | 0.88±0.30 | 1.06±0.39 |
| O2-LE | 10.05±3.14 | 7.86±6.26 | 17.32±26.01 | 7.63±4.33 | 0.80±0.27 | 0.96±0.39 |
| F7-LE | 22.81±16.59 | 6.53±2.30 | 7.03±7.74 | 7.64±8.60 | 2.51±3.93 | 3.86±5.90 |
| F8-LE | 25.24±21.90 | 8.00±4.41 | 8.02±8.60 | 13.57±13.29 | 5.17±5.90 | 7.65±8.43 |
| T3-LE | 9.12±5.65 | 5.24±4.48 | 5.80±6.24 | 6.93±5.27 | 1.73±1.54 | 3.21±3.07 |
| T4-LE | 8.43±3.65 | 6.38±8.37 | 6.32±7.27 | 7.29±4.44 | 1.70±1.13 | 3.18±2.21 |
| T5-LE | 8.49±3.77 | 8.85±12.84 | 15.02±21.69 | 6.70±5.06 | 0.91±0.54 | 1.22±0.78 |
| T6-LE | 10.51±6.03 | 9.01±12.23 | 27.46±61.13 | 7.78±6.39 | 1.16±1.05 | 1.74±1.98 |
| Fz-LE | 23.78±17.01 | 13.76±9.18 | 16.76±19.58 | 7.04±4.01 | 1.13±0.49 | 1.55±1.36 |
| Cz-LE | 16.08±5.83 | 16.97±19.24 | 20.97±29.10 | 7.68±4.35 | 1.04±0.33 | 1.19±0.52 |
| Pz-LE | 13.47±4.70 | 15.58±20.88 | 22.86±35.40 | 8.50±5.05 | 0.97±0.29 | 1.12±0.47 |

## Supplementary Table 2.4 Waitlist control Group, opened eye, baseline

| Waitlist control group (Eye Closed) (N=9) | | | | | | |
|---|---|---|---|---|---|---|
| Location | Delta | Theta | Alpha | Beta | High Beta | Gamma |
| FP1-LE | 378.51±739.6 | 50.84105±.47 | 9.02±8.60 | 18.30±24.46 | 15.02±29.97 | 11.05±15.16 |
| FP2-LE | 359.98±726.1 | 48.66±103.29 | 8.87±8.12 | 21.78±28.73 | 16.96±36.09 | 14.45±20.59 |
| F3-LE | 51.99±72.71 | 13.27±11.6 | 7.41±4.32 | 9.50±4.60 | 3.09±2.67 | 2.78±1.71 |
| F4-LE | 50.54±76.71 | 13.02±12.7 | 7.28±4.21 | 10.78±7.28 | 4.06±5.54 | 3.55±3.35 |
| C3-LE | 21.89±16.03 | 8.68±4.14 | 8.14±5.34 | 10.87±6.72 | 1.97±1.00 | 2.01±0.77 |
| C4-LE | 20.94±15.92 | 7.82±3.85 | 7.93±5.47 | 10.20±6.10 | 1.85±0.87 | 1.67±0.55 |
| P3-LE | 15.43±7.13 | 6.87±2.70 | 9.03±7.49 | 10.88±6.78 | 1.47±0.74 | 1.78±1.37 |
| P4-LE | 15.70±6.97 | 6.35±2.41 | 8.19±7.26 | 8.98±4.20 | 1.12±0.44 | 1.16±0.42 |
| O1-LE | 13.29±5.62 | 5.56±1.72 | 8.50±6.66 | 8.61±4.46 | 1.09±0.39 | 1.53±0.89 |
| O2-LE | 12.88±6.15 | 5.07±1.52 | 6.37±4.29 | 7.46±3.66 | 1.02±0.56 | 1.50±1.12 |
| F7-LE | 57.47±76.05 | 10.23±10.4 | 4.51±2.22 | 8.14±3.97 | 3.04±2.56 | 4.12±3.67 |
| F8-LE | 41.64±62.32 | 7.61±8.26 | 4.02±2.51 | 9.00±4.52 | 3.48±2.60 | 4.58±3.08 |
| T3-LE | 14.16±11.67 | 4.67±2.45 | 4.39±2.18 | 11.06±7.11 | 3.15±2.89 | 5.40±5.42 |
| T4-LE | 12.77±10.60 | 3.77±1.99 | 3.48±2.61 | 6.74±2.09 | 1.64±0.59 | 2.92±1.47 |
| T5-LE | 11.33±6.07 | 4.74±1.85 | 5.89±3.61 | 8.14±4.05 | 1.40±0.94 | 2.17±2.04 |
| T6-LE | 11.43±6.37 | 4.01±1.98 | 7.01±8.02 | 7.33±3.71 | 1.27±0.72 | 2.16±1.67 |
| Fz-LE | 63.14±88.60 | 15.8714.±2 | 9.58±6.89 | 11.42±9.93 | 3.61±4.51 | 6.63±14.76 |
| Cz-LE | 23.48±13.15 | 10.28±4.33 | 8.80±6.05 | 10.33±6.66 | 2.34±1.88 | 1.58±0.58 |
| Pz-LE | 17.30±7.25 | 7.62±2.81 | 9.08±6.97 | 10.12±5.89 | 1.26±0.58 | 1.19±0.39 |

## Supplementary Table 2.5 NSRT Group, Closed eye, Visit 16

| Experimental group (Eye Closed) (N=10) | | | | | | |
|---|---|---|---|---|---|---|
| Location | Delta | Theta | Alpha | Beta | High Beta | Gamma |
| FP1-LE | 14.143254±7.692405 | 7.647461±4.033185 | 21.461333±21.286363 | 6.280375±3.423844 | 1.06401±0.569747 | 1.573161±1.265446 |
| FP2-LE | 14.78042±8.207477 | 8.089589±4.38837 | 21.656886±21.877735 | 6.789822±4.448466 | 1.186407±0.990748 | 1.523869±1.161988 |
| F3-LE | 14.058005±6.592148 | 13.475446±9.521705 | 31.299559±31.204813 | 8.771515±6.044188 | 1.17511±0.610525 | 1.529598±1.249027 |
| F4-LE | 14.535566±6.689705 | 15.231811±12.001828 | 31.075507±29.418427 | 9.277463±7.061521 | 1.155392±0.712943 | 1.460545±0.972993 |
| C3-LE | 13.389863±7.269929 | 15.368206±17.570384 | 31.370076±26.853826 | 9.769476±6.724188 | 1.146058±0.731198 | 1.115654±0.650268 |
| C4-LE | 13.027781±6.797019 | 17.491002±24.825238 | 29.104238±22.515262 | 9.898576±6.264099 | 1.119094±0.629846 | 1.22095±0.750827 |
| P3-LE | 12.640061±6.928852 | 17.475121±28.046903 | 33.788048±29.139672 | 12.430633±10.265592 | 1.020541±0.570405 | 0.917509±0.407397 |
| P4-LE | 13.216132±7.215181 | 17.894218±28.984659 | 36.275456±30.937446 | 11.928131±8.24038 | 1.014194±0.487305 | 0.922387±0.417761 |
| O1-LE | 11.767668±7.333738 | 14.108324±21.818722 | 40.25791±30.326026 | 12.245019±8.815199 | 0.934414±0.44642 | 0.986943±0.477506 |
| O2-LE | 11.005752±7.196534 | 10.885413±11.560595 | 38.821653±33.261811 | 11.66206±7.597613 | 0.930911±0.41636 | 0.979422±0.446212 |
| F7-LE | 15.5599±7.258379 | 6.953004±3.338688 | 16.882362±16.901374 | 5.719545±3.281335 | 0.921099±0.602749 | 1.22253±0.836398 |
| F8-LE | 15.277057±7.497799 | 7.613268±4.517291 | 15.50405±14.752088 | 6.158567±4.531528 | 0.923049±0.646631 | 1.282693±1.028677 |
| T3-LE | 9.673874±7.656003 | 5.865432±5.130949 | 9.951291±7.875592 | 5.957528±4.863125 | 0.912807±0.753728 | 1.616753±1.662436 |

| | | | | | | |
|---|---|---|---|---|---|---|
| T4-LE | 8.795455±7.115217 | 6.875573±9.22567 | 8.972572±6.562575 | 6.114702±6.922079 | 1.04987±1.318796 | 1.629675±2.058621 |
| T5-LE | 9.512484±6.486641 | 11.530563±17.901581 | 24.306048±28.160051 | 8.329332±6.313917 | 0.775075±0.426583 | 0.838305±0.448368 |
| T6-LE | 11.352086±8.35616 | 12.073296±19.713624 | 28.464701±20.557428 | 8.171724±4.967609 | 0.855321±0.536688 | 0.971888±0.736305 |
| Fz-LE | 15.216273±6.633855 | 16.252513±11.956773 | 35.993783±35.48215 | 9.276158±6.137318 | 1.129914±0.518791 | 1.418095±1.20066 |
| Cz-LE | 15.715401±6.517431 | 20.270204±23.080187 | 40.171778±34.339971 | 10.694242±6.769975 | 1.176072±0.577805 | 1.151774±0.472839 |
| Pz-LE | 14.593658±7.018339 | 20.523245±32.651293 | 41.52671±36.155004 | 12.302733±8.836563 | 1.025138±0.491959 | 0.934088±0.38068 |

## Supplementary Table 2.6 Waitlist Control Group, Closed eye, Visit 16

| Waitlist control group (Eye Closed) (N=9) | | | | | | |
|---|---|---|---|---|---|---|
| Location | Delta | Theta | Alpha | Beta | High Beta | Gamma |
| FP1-LE | 42.68077±66.396202 | 13.176747±15.480345 | 24.044859±12.991065 | 7.299208±2.332897 | 1.406141±0.838723 | 2.018812±1.454289 |
| FP2-LE | 41.053343±61.187979 | 12.870472±13.414529 | 24.529713±13.629342 | 8.228693±3.722595 | 1.957218±1.998877 | 3.086162±3.543882 |
| F3-LE | 20.101663±11.150507 | 12.904124±4.603694 | 33.260215±16.581739 | 10.596744±3.656891 | 1.786061±0.863761 | 2.008931±0.819456 |
| F4-LE | 19.918445±11.261126 | 12.9541±4.630188 | 33.520165±19.2465 | 10.42578±4.099775 | 1.669689±0.938261 | 1.918408±0.942363 |
| C3-LE | 16.735072±6.874776 | 11.827963±4.593787 | 34.887917±16.943381 | 15.132164±9.32101 | 2.054203±1.05867 | 2.243535±1.285303 |
| C4-LE | 17.49716±11.817659 | 11.112477±4.892245 | 34.562484±19.791778 | 13.577702±8.300759 | 1.622731±0.867067 | 1.611808±0.555601 |
| P3-LE | 15.269344±6.041211 | 11.310569±4.861279 | 47.260302±40.536154 | 17.747979±12.419991 | 1.605455±1.01117 | 1.888899±1.619933 |
| P4-LE | 15.847872±8.246194 | 11.644967±6.998125 | 53.278308±74.430707 | 15.143563±8.92016 | 1.312326±0.643678 | 1.392201±0.646645 |
| O1-LE | 16.272968±9.703296 | 12.196385±6.110851 | 73.310359±89.564735 | 16.202237±11.057471 | 1.297266±0.510836 | 1.711083±0.881562 |
| O2-LE | 12.514064±5.896314 | 9.861395±4.976425 | 48.920582±66.782091 | 12.376059±7.086631 | 1.212282±0.587154 | 1.663855±1.00159 |
| F7-LE | 17.358618±13.047762 | 7.710269±2.905499 | 18.329749±8.61302 | 7.303801±3.151014 | 1.738281±1.518547 | 2.470329±1.971066 |
| F8-LE | 15.253606±14.234546 | 6.915335±3.63258 | 16.701751±10.264048 | 6.004828±2.543338 | 1.140141±0.593249 | 1.692726±1.164504 |
| T3-LE | 9.591431±3.633702 | 5.625215±1.785191 | 12.790684±4.679947 | 9.837426±6.73361 | 1.985643±2.687594 | 3.572082±4.99722 |
| T4-LE | 8.825034±4.209547 | 5.003304±2.534971 | 12.039768±6.244243 | 6.446952±2.788194 | 1.037169±0.609449 | 1.744596±1.53961 |
| T5-LE | 11.3084±3.769415 | 8.267293±3.183905 | 30.947115±23.105584 | 11.874104±6.537966 | 1.617618±1.907912 | 2.356744±3.437156 |
| T6-LE | 14.752079±13.905319 | 10.479514±10.637453 | 57.314105±90.976387 | 12.470161±10.993075 | 1.296644±0.836194 | 1.867987±1.462588 |

| | | | | | | |
|---|---|---|---|---|---|---|
| Fz-LE | 23.721336±12.917721 | 15.829301±5.350104 | 39.462754±20.076507 | 15.010775±13.822975 | 4.073441±7.3255 | 9.538058±23.929113 |
| Cz-LE | 19.954127±7.852769 | 14.628703±5.16857 | 43.995108±24.400329 | 13.919201±7.660237 | 2.319423±1.701389 | 1.747588±0.628986 |
| Pz-LE | 17.151061±7.621182 | 12.94746±5.848455 | 56.596466±49.679606 | 16.245701±10.239442 | 1.446558±0.703419 | 1.456646±0.629492 |

## Supplementary Table 2.7 NSRT Group, opened eye, Visit 16

| Experimental group (Eye Opened) (N=10) | | | | | | |
|---|---|---|---|---|---|---|
| Location | Delta | Theta | Alpha | Beta | High Beta | Gamma |
| FP1-LE | 67.667188±69.896492 | 11.105061±6.892343 | 9.988715±11.559862 | 7.099866±3.070912 | 1.899822±0.939023 | 2.593407±1.609611 |
| FP2-LE | 80.302348±98.739139 | 14.016985±14.104645 | 10.975956±12.488202 | 8.67346±7.229706 | 2.348317±2.196994 | 3.203296±3.271395 |
| F3-LE | 22.929765±17.487964 | 11.622796±7.340645 | 13.930605±16.222845 | 7.744116±5.318556 | 1.651873±1.140994 | 2.504229±2.206739 |
| F4-LE | 22.102671±15.657462 | 13.195824±10.080484 | 15.291787±18.468606 | 8.685341±7.497954 | 1.928241±1.927516 | 2.888176±3.181678 |
| C3-LE | 13.823187±5.885124 | 13.019836±15.169719 | 15.777462±21.65402 | 7.454725±4.52188 | 1.144299±0.572627 | 1.347584±0.862053 |
| C4-LE | 13.412077±5.143729 | 14.830806±20.44789 | 16.344499±21.45215 | 7.979513±4.168394 | 1.217816±0.521156 | 1.642514±1.145664 |
| P3-LE | 12.004564±3.93623 | 14.33466±21.298071 | 20.920625±31.277379 | 8.753156±7.027564 | 0.92967±0.405375 | 0.96693±0.339639 |
| P4-LE | 12.446448±5.013135 | 13.560013±19.046437 | 22.191835±33.495377 | 8.56592±5.535603 | 0.929513±0.35372 | 1.03981±0.391265 |
| O1-LE | 10.918744±4.029207 | 10.323832±12.55967 | 25.502155±48.050308 | 8.386282±5.471353 | 0.884686±0.301907 | 1.059132±0.392212 |
| O2-LE | 10.050416±3.144292 | 7.855185±6.260103 | 17.319887±26.005874 | 7.628314±4.32562 | 0.80438±0.268349 | 0.964278±0.390377 |
| F7-LE | 22.812953±16.589467 | 6.528101±2.301972 | 7.033651±7.744089 | 7.639085±8.601888 | 2.512193±3.931169 | 3.857467±5.897731 |
| F8-LE | 25.24142±21.900256 | 7.997105±4.411628 | 8.017598±8.596382 | 13.572245±13.292096 | 5.173039±5.900094 | 7.652979±8.433909 |
| T3-LE | 9.122977±5.648129 | 5.238777±4.479539 | 5.804368±6.238938 | 6.927615±5.269607 | 1.734207±1.542959 | 3.208118±3.068016 |
| T4-LE | 8.426471±3.646162 | 6.377716±8.372612 | 6.31524±7.272802 | 7.291579±4.435209 | 1.70235±1.13482 | 3.183967±2.207417 |
| T5-LE | 8.486402±3.767393 | 8.847824±12.837479 | 15.019414±21.69344 | 6.703965±5.06042 | 0.913991±0.538064 | 1.215284±0.77747 |
| T6-LE | 10.508771±6.031825 | 9.005591±12.234486 | 27.459838±61.126461 | 7.77575±6.393838 | 1.156266±1.048976 | 1.739427±1.977204 |
| Fz-LE | 23.783079±17.0 | 13.75985±9.175 | 16.764854±19.5 | 7.035336±4.005 | 1.13479±0.491 | 1.553695±1.36 |

| | | | | | | |
|---|---|---|---|---|---|---|
| | 13617 | 025 | 75683 | 872 | 632 | 4817 |
| Cz-LE | 16.084806±5.83231 | 16.965102±19.244433 | 20.969888±29.102495 | 7.677437±4.354867 | 1.04415±0.332462 | 1.194184±0.5192 |
| Pz-LE | 13.47006±4.702239 | 15.577053±20.881457 | 22.864585±35.395668 | 8.503187±5.050189 | 0.968693±0.290985 | 1.1172±0.467716 |

## Supplementary Table 2.8 Waitlist control Group, opened eye, Visit 16

| Waitlist control group (Eye Closed) (N=9) | | | | | | |
|---|---|---|---|---|---|---|
| Location | Delta | Theta | Alpha | Beta | High Beta | Gamma |
| FP1-LE | 378.51265739.614949 | 50.842327±105.471691 | 9.017066±8.597391 | 18.295832±24.456834 | 15.022915±29.971044 | 11.045628±15.162322 |
| FP2-LE | 359.984763±726.130249 | 48.663666±103.287554 | 8.867182±8.120998 | 21.782814±28.730153 | 16.956178±36.088097 | 14.453732±20.591999 |
| F3-LE | 51.9888±72.712051 | 13.274419±11.624474 | 7.405104±4.324663 | 9.502839±4.60352 | 3.094315±2.669213 | 2.780208±1.714866 |
| F4-LE | 50.538231±76.711346 | 13.021347±12.787776 | 7.284114±4.209328 | 10.776677±7.27664 | 4.060197±5.542679 | 3.549131±3.347651 |
| C3-LE | 21.887468±16.029269 | 8.676014±4.142763 | 8.138709±5.342414 | 10.867874±6.715508 | 1.974768±1.001918 | 2.01078±0.773394 |
| C4-LE | 20.939826±15.923635 | 7.82266±3.850769 | 7.931861±5.472182 | 10.1962±6.101697 | 1.847572±0.872964 | 1.665161±0.552085 |
| P3-LE | 15.427389±7.132993 | 6.873768±2.695024 | 9.031838±7.487115 | 10.883829±6.779629 | 1.468503±0.74197 | 1.782825±1.365533 |
| P4-LE | 15.704309±6.971566 | 6.349259±2.409144 | 8.193747±7.255171 | 8.980488±4.199833 | 1.115875±0.438029 | 1.156959±0.419042 |
| O1-LE | 13.293722±5.620097 | 5.556133±1.721159 | 8.502626±6.664789 | 8.613849±4.455926 | 1.088222±0.389503 | 1.532643±0.887539 |
| O2-LE | 12.881062±6.149537 | 5.073612±1.515645 | 6.373816±4.293838 | 7.456414±3.659022 | 1.023263±0.563286 | 1.499541±1.123218 |
| F7-LE | 57.467811±76.053043 | 10.233943±10.459405 | 4.507176±2.215836 | 8.136598±3.973032 | 3.036686±2.557668 | 4.115348±3.671166 |
| F8-LE | 41.643661±62.31593 | 7.609133±8.263882 | 4.022697±2.513708 | 8.999085±4.521373 | 3.48247±2.595395 | 4.575198±3.083538 |
| T3-LE | 14.16282±11.673102 | 4.670837±2.449122 | 4.393942±2.17714 | 11.063849±7.112388 | 3.148046±2.89284 | 5.397376±5.415934 |
| T4-LE | 12.769229±10.597867 | 3.769361±1.990773 | 3.477954±2.607726 | 6.740608±2.09013 | 1.638882±0.591467 | 2.924431±1.469582 |
| T5-LE | 11.326289±6.067047 | 4.743204±1.847316 | 5.893874±3.611945 | 8.138483±4.04681 | 1.395443±0.942892 | 2.171807±2.042922 |
| T6-LE | 11.426256±6.37177 | 4.014527±1.975473 | 7.005055±8.017455 | 7.326432±3.712027 | 1.268313±0.716182 | 2.158316±1.666762 |
| Fz-LE | 63.143169±88.60 | 15.871702±14.24 | 9.582886±6.8 | 11.419657±9.92 | 3.609416±4.506 | 6.630704±14.75 |

| | | | | | | |
|---|---|---|---|---|---|---|
| | 0936 | 6265 | 93323 | 8002 | 569 | 8339 |
| Cz-LE | 23.484641±13.152662 | 10.281423±4.333497 | 8.801915±6.049975 | 10.325832±6.655894 | 2.339882±1.878608 | 1.577593±0.579112 |
| Pz-LE | 17.304222±7.251098 | 7.618804±2.813701 | 9.083776±6.971124 | 10.115552±5.88604 | 1.264182±0.578169 | 1.194712±0.387113 |
